# Challenges in implementing the 2015 BASHH guidelines for the appropriate use of post-exposure prophylaxis for HIV following sexual exposure

**DOI:** 10.12688/f1000research.8952.1

**Published:** 2016-06-09

**Authors:** Mohammad Fawad Khattak

**Affiliations:** 1 Department of Medicine, Walsall Manor Hospital, Health Education, West Midlands, WS2 9PS, UK

**Keywords:** HIV, post exposure prophylaxis for HIV, BASHH, antiretrovirals, sexually transmitted infections

## Abstract

The use of post exposure prophylaxis for human immunodeficiency virus (HIV) following sexual exposure (PEPSE) was retrospectively audited in an inner city genitourinary clinic against the 2015 national guidelines by the British Association for Sexual Health and HIV (BASHH). One-hundred out of a total of 101 patients (99%) had a baseline HIV test done. 82.1% (n=83) of patients were given PEPSE prescriptions fitting within recommended indications lower than the 90% target set by BASHH. 84.2% (n=85) of patients had PEPSE administered within 72 hours lower than the 90%. 61.4% (n=62) of patients were known to have completed four weeks of PEPSE lower than the 75% target. 61.4% (n=62) of patients were screened for sexually transmitted infections (STIs) lower than the 90% target. 59.4% (n=60%) of patients had post-PEPSE HIV bloods slightly lower than the 60% target.

## Introduction

Post-exposure prophylaxis for HIV involves taking antiretrovirals by human immunodeficiency virus (HIV)-negative individuals for four weeks, after a suspected or known exposure to HIV to reduce the risk of transmission ^1, 2^. In 2015 the British Association for Sexual Health and HIV updated national guidelines on the appropriate use of post exposure prophylaxis after sexual exposure to HIV (PEPSE) ^3^. -- The guidelines provide indications for when PEPSE use; is recommended; can be considered; or is not appropriate. It also recommends PEPSE use within 72 hours; baseline HIV testing, appropriate sexually transmitted infection (STI) testing, and completion of four weeks of PEPSE with follow up HIV bloods after completion of PEPSE. BASHH have specified auditable targets for these recommendations, and this retrospective audit compares the use of PEPSE in our genitourinary clinic against these recommendations.

## Method

A retrospective case note review was carried out at Walsall Centre of Sexual Health. One-hundred one patients who were coded as having received PEPSE between June 2013 and September 2015 were identified on the computer system. No permission was required to conduct the study and publish these results. Notes of these patients were reviewed and data regarding; the indication of PEPSE administration; time since exposure; investigations carried out; completion of four weeks of PEPSE and whether the patient had follow up investigation were uploaded onto a Microsoft Excel database.

## Results

The results of 101 patients who received PEPSE were analysed ( Table 1). 48.5% (n=49) of patients were male, 61% (n=30) of the male patients were bi-sexual/homosexual.

**Table 1.** Auditable targets for prophylaxis for human immunodeficiency virus (HIV) following sexual exposure.

Patients			
	Number	%	BASHH recommended target %
**Baselines HIV Test**	100	99	100
**Indication**			
Recommended Consider	53 30	52.5 29.7	90
Not Recommended	13	12.9	
No documentation	5	5.0	
**Exposure to PEP Time**			
<72 hours	85	84.2	90
>72 hours	11	10.9	
Not documented	5	5	
**Completion of 4** **weeks of PEPSE**			
Yes	62	61.4	75
No	14	13.9	
Unknown	25	24.8	
**STI Screening**			
Hep B immunity/booster /Syphilis	101	100	
Chlamydia/gonorrhoea	62	61.4	90
**Post-PEPSE HIV Test**			
	60	59.4	60

Baseline HIV tests were done in 99% of patients (n=100). One patient did not have baseline HIV tests. This patient initially visited a local emergency department where she received PEPSE without HIV testing. This patient subsequently came to our genitourinary medicine clinic one week later and had a HIV test done. Baseline HIV test was done on the first visit to the clinic in all 100 cases.

52.5% (n=53/101) of prescriptions for PEPSE were given under recommended indications by BASHH ( Table 2), and 29.7% (n=30/101) of patients were given PEPSE under indications where BASHH state they can be considered. All of the patients in the considered category in this audit were female patients who had been sexually abused. In total, 82.2% of patients were given PEPSE for recommended/considered indications lower than the 90% target. 5% (n=5/101) of patients had no documented reason for starting PEPSE.

**Table 2.** Patients that fit into recommended indications (n=83).

	Number of patients	%
**Patients that fit recommended** **Indications**		
HIV +ve partner VL Unknown or >40	20	24.1
Men who have sex with men (MSM) High prevalence/ unknown	18	21.7
Sexual Assault	31	37.3
Needle stick Injury	15	18.1

84.2% (n=85) of patients received PEPSE within 72 hours of exposure lower than the 90% target. 13.9% (n=14) of PEPSE was prescribed after 72 hours since exposure, while 5% (n=5) of patients had no documentation of when the exposure occurred.

61.4% (n=62) of patients had documentation showing that they had completed four weeks of PEPSE, lower than the 75% target. 13.9% (n=14) of patients did not complete four weeks of PEPSE while 24.8% (n=25) of patients had no documentation regarding whether they had completed four weeks of PEPSE.

59.4% (n=60%) of patients had post-PEPSE HIV bloods slightly lower than the 60% target.

All 100 patients that had baseline HIV bloods taken had appropriate investigations into hepatitis B and syphilis. However 61.4% (n=62) of patients were screened for chlamydia and gonorrhoea lower than the 90% target. None of the patients who had come for PEPSE after needle stick injury had testing for gonorrhoea and chlamydia, and not taking into account these patients 73% of patients had screening for chlamydia and gonorrhoea.

## Discussion

The majority of patients prescribed PEPSE where given so under indications that were deemed compliant with BASHH guidelines and within 72 hours of the suspected exposure. All but one of these patients had baselines HIV bloods taken, and appropriate testing for syphilis and hepatitis B, with post-PEPSE follow-up testing levels being near the BASHH target.

Documentation regarding whether patients were taking or discontinuing PEPSE was lacking. There was also difficulty determining whether patients who did not attend after their initial visit had completed their PEPSE course.

One particular guideline that we found difficulty in reaching was screening for chlamydia and gonorrhoea, especially in patients coming in after needlestick injuries. Often times it is either not considered suitable or the patient declines the screening as they do not feel they are at risk. Screening should always be encouraged and there should be documentation that the screening tests have been declined if that is case.

Another issue is patients not visiting for follow-ups. Often screening for chlamydia and gonorrhoea is delayed until after the window period for investigations to identify these organisms. Follow-ups are also important for identifying whether the patient is compliant with the antiretrovirals, and for post-PEPSE HIV blood tests.

Carrying out an audit against the BASHH guidelines have highlighted areas in our clinical practice which need improvement. In response to this audit we have created clear proformas for prescribing PEPSE which include; whether the indication the patient is coming in with fits with BASHH guidelines; whether the exposure was less than 72 hours ago, and a list of the relevant investigations that should be considered. Proformas for follow-up have also been made to assess whether the patient is completing the course of PEPSE and having follow-up bloods after completion of therapy. Training has been given to educate all staff on the indications for PEPSE prescribing, the need to identify HIV status and viral load of source, the need to have accurate documentation, to offer rapid-HIV testing, and fourth generation HIV testing for post-PEPSE follow-up. We have also decided to get patients to book their follow-up appointment during their initial visit to the clinic. We will then send SMS reminders the day before the follow-up appointment to remind patients to attend. We plan to carry out a re-audit in one year.

## Data availability

The data referenced by this article are under copyright with the following copyright statement: Copyright: © 2016 Khattak MF

All raw data are provided in the tables above.
