# Supplementary material for: Challenges in implementing the 2015 BASHH guidelines for the appropriate use of post-exposure prophylaxis for HIV following sexual exposure
Source: F1000Res. 2016 Jun 9;5:1313. [Version 1] doi: 10.12688/f1000research.8952.1 (PMC5464221; doi:10.12688/f1000research.8952.1)
Supplement: Peer review history

# Peer review: Referee response for version 1

**DOI:** 10.5256/f1000research.9631.r23142

**Published:** 2017-06-07

**Authors:** Rachel Beanland

**Affiliations:** 1 Independent Public Health Specialist, Gaillard, France

This is an interesting report of a clinical audit conducted to assess the implementation of UK BASHH guidelines on Post Exposure Prophylaxis following sexual exposure. The paper provides a clear summary of the methods and results compared to national guidelines. The recommendations and subsequent actions taken following the results are of interest to the audience. Further clarification in some sections could enhance the key message of the paper. In places the grammar and language could be improved. 

 Comments and considerations for the authors: 

 Method 

- Please provide further geographical detail (region, country) to allow the audience to understand the context of the setting. 
- Consider rewording the sentence regarding permission. As secondary data has been used no patient consent was needed but how was confidentiality maintained, was the data anonymised? Did you seek any ethical approval or institutional approval? 
- Note Excel is not a classic database, please revise to spreadsheet, unless alternative database was used for analysis. 

Results 

- Consider using 'performed' or 'conducted' in place of 'done'. 
- Table 2. The article focuses on prophylaxis from sexual exposure, it is not clear why needle stick injury is included in this data set. If this is the case, please specify why included. Please review the data presented in the table for accuracy. 
- Do you have any detail on the ARVs prescribed and how adherent the individuals were? 

References 

 Please review the references and provide uptodate citations. WHO PEP guidelines http://apps.who.int/iris/bitstream/10665/145719/1/9789241508193_eng.pdf  the supporting evidence Clinical Infectious Disease supplement https://academic.oup.com/cid/issue/60/suppl_3 be helpful.

I have read this submission. I believe that I have an appropriate level of expertise to confirm that it is of an acceptable scientific standard, however I have significant reservations, as outlined above.

# Peer review: Referee response for version 1

**DOI:** 10.5256/f1000research.9631.r22154

**Published:** 2017-05-26

**Authors:** Paul Volberding

**Affiliations:** 1 UCSF-GIVI Center for AIDS Research, University of California, San Francisco, San Francisco, CA, 94121, USA

This is an interesting report of the use of post-exposure prophylaxis following possible sexual exposure to HIV in a UK retrospective cohort. While it shows that British guidelines were generally followed well, it of course leaves open the question of what effect the prophylaxis had in terms of eventual HIV seroconversion. Another question raised is how many of those receiving PEP in this study may have also been appropriate candidates for PeEP given our current knowledge of the effectiveness of that prevention approach.

I have read this submission. I believe that I have an appropriate level of expertise to confirm that it is of an acceptable scientific standard.

# Peer review: Referee response for version 1

**DOI:** 10.5256/f1000research.9631.r22009

**Published:** 2017-04-20

**Authors:** Theresa Marié Rossouw

**Affiliations:** 1 Department of Immunology, Institute for Cellular and Molecular Medicine, University of Pretoria, Pretoria, South Africa

There are some language and grammatical errors that need attention.

 The link provided to the BASHH guidelines is for the draft document and not the final version.

 More detail about the context is need e.g. where is the Walsall Centre of Sexual Health located?

 Reference 1: I could unfortunately not access the full article, but the reference to this 1997 article in support of post-exposure prophylaxis does not seem to be the best choice since it deals with "Immune response to human immunodeficiency virus (HIV) in health care workers occupationally exposed to HIV-contaminated blood" and does not seem to deal with post-exposure prophylaxis. 

 The second reference in support of PEP is also not the most relevant reference since it is an animal study and the final conclusion of this article is that “limited antiviral drug diffusion in secondary lymphoid tissues may allow persistent viral replication in these tissues and could represent an obstacle to HIV prevention and eradication”.

 It would be interesting to know how many of the patients were repeat users of PEPSE. A possible recommendation could be monitoring for repeat users of PEPSE and referring them for PrEP.

 It would also be interesting to know what antiretroviral medication had been prescribed for PEPSE and whether this complied with the guidelines.  

 It is preferable not to refer to "bi-sexual/homosexual" but rather to men who have sex with men and/or women.

 Table 1:

 Under “Baselines HIV Test” and “Post-PEPSE HIV Test”, grammar and placing of results should receive attention.

 Table 2 needs more explanation since the numbers do not add up. For instance, the text shows that 53/101 had a recommendation for PEPSE, whereas the table shows only 20 HIV+ with unknown or high VL and 18 MSM. The text shows that 30/101 had circumstances under which PEPSE could be considered but the table shows 31 cases of sexual assault. There were 15 cases of needle stick injury. This seems to be very high and since the guideline only covers needle stick injuries in the community, the circumstances surrounding the injuries should be explained. I recommend that all exposure categories be listed in order to improve understanding. It seems that Tables 1 and 2 could be merged.

 Table 2: The recommended VL above which PEPSE should be given is 200 copies/ml and not 40 copies/ml – the unit of measure is not given in the table. In addition, the meaning of the category “Men who have sex with men (MSM) High prevalence/unknown” is unclear.

 The proportion of PEPSE prescriptions administered within 24 hours (and not 72 hours) of risk exposure is the auditable outcome and should also be shown.

 The author states that “There was also difficulty determining whether patients who did not attend after their initial visit had completed their PEPSE course”. What measures were taken to determine this?

 What steps had been taken to assess the VL of HIV-infected partners?

 “No permission was required to conduct the study and publish these results.” I presume the author means that informed consent was not considered relevant since the data used were anonymous, but surely institutional permission was needed?

 Article should make reference to other related work, e.g.

 Ladipo Z, Chauhan M, Foster K. Survey of PEPSE provision: lessons to be learnt. *Sex Transm Infect *2015; **91:**164 ^1^.

 Currie S, Shafiq V, Mellor J *, et al*. P73 The source of the problem – re-audit of pepse provision at an inner city sexual health clinic. *Sex Transm Infect *2015; **91:**A39-A40 ^2^

 Raha D; Gilson R. An audit of PEPSE in an integrated sexual and reproductive health centre resulting in change of local protocol in accordance to national guidelines: room for improvement. HIV Medicine. 17():33–34, Apr 2016 ^3^

I have read this submission. I believe that I have an appropriate level of expertise to confirm that it is of an acceptable scientific standard, however I have significant reservations, as outlined above.

## References

[1] LadipoZChauhanMFosterK: Survey of PEPSE provision: lessons to be learnt. *Sexually Transmitted Infections*.2015;91(3) : 10.1136/sextrans-2014-051933 10.1136/sextrans-2014-051933 25887852

[2] CurrieSShafiqVMellorJMcCordJHoustonJBabuC: P73 The source of the problem – re-audit of pepse provision at an inner city sexual health clinic. *Sexually Transmitted Infections*.2015;91(Suppl 1) : 10.1136/sextrans-2015-052126.116 A39.3-A40 10.1136/sextrans-2015-052126.116 25609465

[3] RahaDGilsonR: An audit of PEPSE in an integrated sexual and reproductive health centre resulting in change of local protocol in accordance to national guidelines: room for improvement. *HIV Medicine*.2016;17:33-34
